# The impact of the roll-out of rapid molecular diagnostic testing for tuberculosis on empirical treatment in Cape Town, South Africa

**DOI:** 10.2471/BLT.16.185314

**Published:** 2017-04-28

**Authors:** Sabine Hermans, Judy Caldwell, Richard Kaplan, Frank Cobelens, Robin Wood

**Affiliations:** a The Desmond Tutu HIV Centre, Institute for Infectious Diseases and Molecular Medicine, University of Cape Town Faculty of Health Sciences, Anzio Road, Observatory, Cape Town, 7925, South Africa.; b City of Cape Town Health Directorate, Cape Town, South Africa.; c Amsterdam Institute for Global Health and Development, Amsterdam, Netherlands.

## Abstract

**Objective:**

To investigate the impact of introducing a rapid test as the first-line diagnostic test for drug-sensitive tuberculosis in Cape Town, South Africa.

**Methods:**

Xpert® MTB/RIF (Xpert®), an automated polymerase-chain-reaction-based assay, was rolled out between 2011 and 2013. Data were available on 102 007 adults treated for pulmonary tuberculosis between 2010 and 2014. Tuberculosis notification rates per 100 000 population were calculated for each calendar year and for each year relative to the test roll-out locally, overall and by bacteriological confirmation. Empirical treatment was defined as treatment given without bacteriological confirmation by Xpert®, sputum smear microscopy or sputum culture.

**Findings:**

Between 2010 and 2014, the proportion of human immunodeficiency virus (HIV)-negative patients treated empirically for tuberculosis declined from 23% (2445/10 643) to 11% (1149/10 089); in HIV-positive patients, it declined from 42% (4229/9985) to 27% (2364/8823). The overall tuberculosis notification rate decreased by 12% and 19% among HIV-negative and HIV-positive patients, respectively; the rate of bacteriologically confirmed cases increased by 1% and 3%, respectively; and the rate of empirical treatment decreased by 56% and 49%, respectively. These changes occurred gradually following the test’s introduction and stabilized after 3 years.

**Conclusion:**

Roll-out of the rapid test in a setting with a high prevalence of pulmonary tuberculosis and HIV infection was associated with a halving of empirical treatment that occurred gradually after the test’s introduction, possibly reflecting the time needed for full implementation. More than a quarter of HIV-positive patients with tuberculosis were still treated empirically, highlighting the diagnostic challenge in these patients.

## Résumé

**Objectif:**

Analyser l'impact de l'introduction d'un test rapide comme test initial de diagnostic de la tuberculose pharmacosensible au Cap, en Afrique du Sud.

**Méthodes:**

Le test Xpert® MTB/RIF (Xpert®), un test automatisé qui repose sur le principe de l'amplification en chaîne par polymérase, a été déployé entre 2011 et 2013. Des données étaient disponibles au sujet de 102 007 adultes traités contre la tuberculose pulmonaire entre 2010 et 2014. Le taux de signalement de cas de tuberculose pour 100 000 habitants a été calculé pour chaque année civile et pour chaque année de déploiement du test à l'échelon local, global et avec confirmation bactériologique. Nous avons défini les traitements empiriques comme les traitements donnés sans confirmation bactériologique par le test Xpert®, examen microscopique de frottis d'expectorations ou culture d'expectorations.

**Résultats:**

Entre 2010 et 2014, la proportion de patients séronégatifs au virus de l'immunodéficience humaine (VIH) traités empiriquement contre la tuberculose est passée de 23% (2445/10 643) à 11% (1149/10 089); chez les patients séropositifs, elle est passée de 42% (4229/9985) à 27% (2364/8823). Le taux global de signalement des cas de tuberculose a baissé respectivement de 12% et de 19% chez les patients séronégatifs et séropositifs au VIH; le taux confirmé bactériologiquement a augmenté respectivement de 1% et de 3%; et le taux d’administration de traitements empiriques a baissé respectivement de 56% et de 49%. Ces changements sont intervenus progressivement après l'introduction du test et se sont stabilisés au bout de 3 ans.

**Conclusion:**

Le déploiement du test rapide dans une zone ayant une forte prévalence de tuberculose pulmonaire et de VIH a été associé à une réduction de moitié des traitements empiriques, qui s'est faite progressivement après l'introduction du test -ce qui reflète peut-être le temps nécessaire à sa mise en œuvre intégrale. Plus d'un quart des patients séropositifs au VIH atteints de tuberculose étaient encore traités empiriquement, ce qui montre les enjeux du diagnostic pour ces patients.

## Resumen

**Objetivo:**

Investigar el impacto de la introducción de una prueba rápida como la prueba de diagnóstico de primera línea para la tuberculosis sensible a los medicamentos en Ciudad del Cabo, Sudáfrica.

**Métodos:**

Entre 2011 y 2013 se implementó Xpert® MTB/RIF (Xpert®), una prueba automatizada basada en una reacción en cadena de polimerasa. Se dispuso información sobre 102 007 adultos tratados de tuberculosis pulmonar entre 2010 y 2014. Se calcularon las tasas de notificación de tuberculosis por cada 100 000 habitantes por año civil y por año en relación con la puesta en marcha de la prueba a nivel local, general y por confirmación bacteriológica. El tratamiento empírico se definió como un tratamiento proporcionado sin confirmación bacteriológica por parte de Xpert®, microscopia de frotis de esputo o cultivo de esputo.

**Resultados:**

Entre 2010 y 2014, el porcentaje de pacientes negativos en el virus de la inmunodeficiencia humana (VIH) tratados de forma empírica de tuberculosis cayó de un 23% (2 445/10 643) a un 11% (1 149/10 089); en pacientes positivos en VIH, cayó de un 42% (4 229/9 985) a un 27% (2 364/8 823). En general, la tasa de notificación de tuberculosis cayó un 12% y un 19% entre pacientes con VIH negativo y positivo, respectivamente; la tasa de casos confirmados a nivel bacteriológico aumentó un 1% y un 3%, respectivamente; y la tasa de tratamiento empírico se redujo un 56% y un 49%, respectivamente. Estos cambios se produjeron de forma gradual tras la implementación de la prueba y se estabilizaron pasados 3 años.

**Conclusión:**

La puesta en marcha de la prueba rápida en un lugar con una alta prevalencia de tuberculosis pulmonar e infección por VIH se asoció con una reducción a la mitad del tratamiento empírico que se aplicó de forma gradual tras la introducción de la prueba, reflejando posiblemente el momento necesario para la completa implementación. Aun así, más de un cuarto de los pacientes positivos en VIH y con tuberculosis se sometieron a un tratamiento empírico, lo que destaca el problema del diagnóstico en estos pacientes.

## ملخص

**الغرض:**

الوقوف على تأثير عملية طرح اختبار سريع كاختبار تشخيصي أولي للسل المقاوم للأدوية في كيب تاون بجنوب أفريقيا.

**الطريقة:**

يمثل Xpert® MTB/RIF (والمعروف اختصارًا باسم Xpert®‎) فحصًا آليًا قائمًا على تفاعل البوليميراز المتسلسل، تم نشره في الفترة ما بين عامي 2011 و2013. وكانت البيانات متاحة عن 102,007 من البالغين الذين تم علاجهم من السل الرئوي في الفترة بين عامي 2010 و2014. وتم حساب معدلات الإبلاغ عن السل لكل شريحة سكانية تبلغ 100,000 نسمة عن كل سنة تقويمية، وعن كل سنة تبعًا لعمليات طرح الاختبار على المستوى المحلي، وبشكل عام، وعن طريق تأكيد البكتيريا. تم تعريف العلاج التجريبي على أنه علاج مقدم دون تأكيد البكتيريا باستخدام اختبار Xpert®‎، أو الفحص المجهري لمسحة البلغم، أو مستنبت البلغم.

**النتائج:**

بين عامي 2010 و2014، انخفضت نسبة غير المصابين بفيروس العوز المناعي البشري (HIV) الذين تلقوا علاجًا تجريبيًا من السل من 23‏٪ (2445/10,643) إلى 11‏٪ (1149/10,089)؛ وفيما يخص المرضى المصابين بفيروس العوز المناعي البشري (HIV)، فقط انخفضت النسبة من 42‏٪ (4229/9985) إلى 27‏٪ (2364/8823). كما انخفض المعدل الإجمالي للإبلاغ عن السل بنسبة ‏12‏٪ و‏‏19٪ بين المرضى غير المصابين بفيروس نقص المناعة البشرية وكذلك بين المصابين بالفيروس على التوالي؛ وارتفع معدل الحالات المؤكدة للبكتريا بنسبة 1‏٪ و‏3‏٪ على التوالي، كما انخفض معدل العلاج التجريبي بنسبة ‏56‏٪ و49‏٪ على التوالي. ظهرت هذه التغيرات تدريجيًا بعد طرح الاختبار وثباته بعد 3 سنوات.

**الاستنتاج:**

ارتبط طرح الاختبار السريع في بيئة ذات معدل انتشار مرتفع للسل الرئوي وعدوى فيروس العوز المناعي البشري (HIV) بخفض العلاج التجريبي الذي حدث تدريجيًا بعد استقدام الاختبار، مما يعكس على الأرجح الوقت اللازم للتنفيذ الكامل. ولا يزال أكثر من ربع المصابين بفيروس العوز المناعي البشري والسل يتلقون علاجًا تجريبيًا، مما يسلط الضوء على التحدي التشخيصي لدى هؤلاء المرضى.

## 摘要

**目的:**

旨在调查在南非开普敦引进一项快速检测作为药物敏感性结核病一线诊断检测方法的影响。

**方法:**

Xpert® MTB/RIF (Xpert®) 是一项基于聚合酶链反应的自动分析项目，于 2011 年至 2013 年间开展。分析数据可用于 2010 年至 2014 年间接受肺结核病治疗的 102 007 名成人。分别针对开展的局部性检测、整体性检测及经细菌学证实的检测计算了每个日历年及每年每 100 000 人口的结核病呈报率。 经验性治疗被定义为未经过 Xpert® 细菌学证实、未进行痰涂片镜检或痰培养的疗法。

**结果:**

在 2010 年至 2014 年间，接受结核病经验性治疗的人类免疫缺陷病毒 (HIV)-阴性患者的比例从 23% (2445/10 643) 降至 11% (1149/10 089)；在 HIV-阴性患者中，该比例从 42% (4229/9985) 降至 27% (2364/8823)。 总体而言，HIV-阴性患者和 HIV-阳性患者的结核病呈报率分别降低 12% 和 19%；经细菌学证实的病例比率分别增加 1% 和 3%；接受经验性治疗的比例分别降低 56% 和 49%。 自该检测方法引进以来，这些变化逐渐发生并在 3 年后稳定下来。

**结论:**

在肺结核病和 HIV 感染高发地区开展快速检测导致引进该检测后经验性治疗逐渐减少了一半，这可能反映出全面推行该项检测所需的时间。 超过四分之一的 HIV-阳性结核病患者仍在接受经验证治疗，这凸显了这些患者面临的诊断方面的挑战。

## Резюме

**Цель:**

Изучить влияние введения экспресс-метода в качестве диагностического теста первой линии на чувствительный к лекарственным средствам туберкулез в Кейптауне, Южная Африка.

**Методы:**

Xpert® MTB/RIF (Xpert®), автоматизированный анализ на основе полимеразной цепной реакции, который был введен в период между 2011 и 2013 годами. Имелись данные, полученные от 102 007 взрослых пациентов, которые лечились от туберкулеза легких в период с 2010 по 2014 год. Показатель регистрируемой заболеваемости туберкулезом (количество случаев на 100 000 человек) был рассчитан для каждого календарного года, а также для каждого года относительно введения теста на местном уровне в целом и с помощью бактериологического подтверждения. Эмпирическое лечение определяли как лечение, проводимое без бактериологического подтверждения с помощью Xpert®, микроскопии мазка мокроты или бактериологического исследования мокроты.

**Результаты:**

В период между 2010 и 2014 годами доля ВИЧ-отрицательных (ВИЧ — вирус иммунодефицита человека) пациентов, получавших эмпирическую терапию при туберкулезе, снизилась с 23% (2445/10 643) до 11% (1149/10 089), у ВИЧ-положительных пациентов этот показатель снизился с 42% (4229/9985) до 27% (2364/8823). Общий уровень заболеваемости туберкулезом снизился на 12 и 19% среди ВИЧ-отрицательных и ВИЧ-положительных пациентов соответственно, частота бактериологически подтвержденных случаев заболеваемости увеличилась на 1 и 3% соответственно, и частота применения эмпирического лечения снизилась на 56 и 49% соответственно. Эти изменения происходили постепенно после введения теста и стабилизировались через 3 года.

**Вывод:**

Введение экспресс-теста в условиях высокой распространенности туберкулеза легких и ВИЧ-инфекции было связано с двукратным сокращением применения эмпирического лечения, которое происходило постепенно после введения теста, что, по-видимому, отражает время, необходимое для полного внедрения. Более четверти ВИЧ-положительных пациентов с туберкулезом по-прежнему лечились эмпирически, что подчеркивает проблемы с диагностикой у этих пациентов.

## Introduction

Sputum smear microscopy is traditionally the first-line diagnostic test for tuberculosis in countries without routine access to the gold standard: sputum culture. This approach is limited by low sensitivity, particularly among patients who test positive for the human immunodeficiency virus (HIV), and is associated with diagnostic delays, underdiagnosis and empirical treatment.^1^ The Xpert® MTB/RIF (Xpert®) test (Cepheid, Sunnyvale, United States of America) is an automated, cartridge-based, rapid molecular diagnostic test for *Mycobacterium tuberculosis* and its resistance to rifampicin.^2^ The test detects the *rpoB* gene of *M. tuberculosis*, including mutations that encode rifampicin resistance, using a real-time polymerase chain reaction and takes less than 2 hours. Because the test has higher sensitivity than smear microscopy (88% versus 65%, respectively),^3^ is rapid and has the ability to detect rifampicin resistance immediately, the World Health Organization (WHO) endorsed its use in resource-constrained settings in December 2010.^4^ By the end of 2014, concessionary pricing had led to widespread roll-out of the test in lower-income countries.^5^

In 2013, WHO identified understanding the impact of the Xpert® test on individual and public health outcomes as one of the top 10 research areas in tuberculosis.^6^ Modelling studies indicated the test would increase tuberculosis case-finding and that the resulting earlier treatment would improve outcomes, leading eventually to reductions in tuberculosis incidence and mortality.^7^^–^^9^ However, the four large randomized trials published to date failed to document these reductions.^10^^–^^13^ This failure may have been due to empirical treatment being replaced by bacteriologically confirmed treatment rather than to more patients being identified.^14^ Subsequently, when one of the original modelling papers was modified to align its results with one of the trials, the predicted decline in tuberculosis incidence decreased from 6% to 1.6%.^10^^,^^15^ Modelling the effect of the test in India produced similar results.^16^

There is a need for more data on the impact of the Xpert® test in practice. In South Africa, roll-out of this test as the primary test in a new tuberculosis diagnostic algorithm started in March 2011 – it was completed in Cape Town in February 2013.^17^ In this study, we evaluated the impact of the test roll-out in Cape Town on the diagnosis of patients with drug-sensitive tuberculosis, stratified by HIV status. We also analysed associated changes in the proportion of notified tuberculosis cases that were confirmed bacteriologically and examined risk factors for empirical treatment. Finally, we determined whether the changes observed increased with time following the introduction of the test.

## Methods

The estimated population of Cape Town in 2011 was 3.7 million.^18^ The diagnosis and treatment of tuberculosis in the city was provided by 101 government primary-care clinics; tuberculosis treatment in private clinics was infrequent.^19^ The diagnosis of pulmonary tuberculosis was generally based on sputum smear microscopy, with sputum culture reserved for patients who remained symptomatic despite negative microscopy findings or who were being retreated. At all clinics, chest X-ray facilities were available, either on-site or through referral. Although the empirical treatment of tuberculosis based on symptoms and chest X-ray findings alone was discouraged, it was an accepted practice for patients who remained symptomatic despite negative microbiological findings.

The Xpert® test machines were installed in all laboratories in Cape Town between August 2011 and February 2013 and use of the test as the primary diagnostic test in the tuberculosis diagnostic algorithm for Western Cape Province was endorsed in a circular to all primary health-care clinics in January 2013.^17^ Two sputum samples were collected and submitted simultaneously to a laboratory – one was for the rapid test. If a rifampicin-sensitive *M. tuberculosis* strain was detected, the second sample was used to determine the sputum smear status pretreatment and, thereby, helps identify smear conversion during follow-up. If a rifampicin-resistant strain was detected, the second sample was used for culture and for testing drug sensitivity. If the first sample from an HIV-infected patient tested negative, the second was used for culture. If the first sample from an HIV-negative patient tested negative, the second was discarded because of the test’s higher sensitivity in these patients. When the test was introduced, the treatment regimen for previously treated patients was changed to that for new patients because rifampicin resistance could then be identified before treatment.^17^

Our population-based study covered 2010 to 2014: 2010 was the last full calendar year before the test roll-out began in Cape Town and 2014 was the first full calendar year after roll-out had been completed. We included all pulmonary tuberculosis patients aged 15 years or older who started treatment during that period. To avoid duplication, we excluded patients transferred between subdistricts. We used anonymized data from the City of Cape Town electronic tuberculosis register on the patients’ characteristics, microbiological test results, chest X-ray results, treatment initiation dates and treatment outcomes. Patients with drug-resistant tuberculosis were entered into a separate register and were not included in our evaluation.

**Fig. 1:**
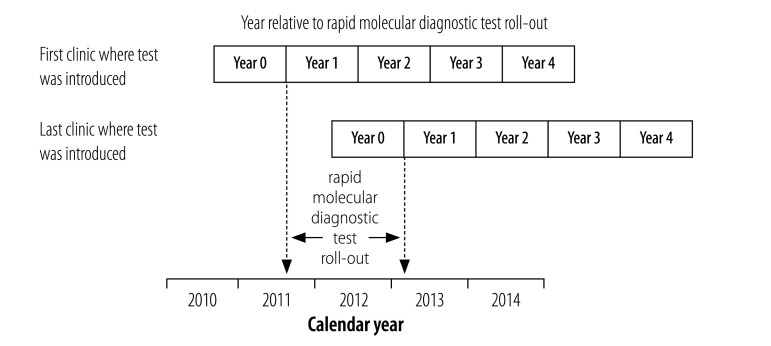
Calendar year and year relative to rapid diagnostic test roll-out, Cape Town, South Africa, 2010–2014

Notes: Patients who initiated tuberculosis treatment in the year before the Xpert® MTB/RIF test (Cepheid, Sunnyvale, United States of America) roll-out date for their clinic were assigned to year 0, those who started treatment in the first year after roll-out for their clinic were assigned to year 1 and so on up to year 4. The Xpert® MTB/RIF test was introduced gradually as the first-line diagnostic test at 101 clinics between August 2011 and February 2013.

We defined the primary method of diagnosis as either: (i) the rapid test; (ii) sputum smear microscopy; or (iii) sputum culture – if more than one test was positive, the primary method was the first test in this sequence. We defined bacteriological confirmation of infection as a positive result to one of these tests. Empirical treatment was defined as treatment given when no test was positive or no test was performed. In addition, patients who tested positive on sputum culture and negative on, or did not undergo, other tests were regarded as having started empirical treatment if their sputum sample was sent for culture after treatment initiation or up to 6 days before initiation (assuming that 7 days was the minimum time required for a positive sputum culture result).^20^ Treatment followed national guidelines and did not differ by method of diagnosis.^21^ Two definitions of time were used: the calendar year and the year relative to the time when the test was introduced in the diagnosing clinic. For the latter year, we identified the date on which the first test result was recorded for each clinic and used this date as the roll-out date for that clinic. Patients were then assigned to a year depending on when their treatment started relative to the test roll-out in their clinic: patients who initiated treatment in the 365 days before the roll-out date were assigned to year 0 and those who started treatment 1096 to 1460 days after the roll-out date were assigned to year 4; other patients were assigned to intermediate years accordingly (Fig. 1). The patient was defined as HIV-positive at the time of tuberculosis treatment if the tuberculosis register recorded: (i) a positive HIV serological test result; (ii) treatment with antiretroviral therapy or co-trimoxazole; or (iii) a CD4+ T-lymphocyte (CD4+ cell) count. The patient was HIV-negative if a negative HIV serological test result had been recorded. All other patients were regarded as having an unknown HIV status.

### Statistical analysis

We used descriptive statistics to present data on patients’ demographic and clinical characteristics in each calendar year and on the primary method of diagnosis in each calendar year and in each year relative to the test roll-out. We calculated annual population disease rates by dividing the total number of bacteriologically confirmed and empirically treated pulmonary tuberculosis patients aged 15 years or older in a year by the mid-year estimate of the adult population in the study area.^22^ Rates were also stratified by bacteriological confirmation and HIV status. The size of the HIV-negative and HIV-positive adult population was derived using annual HIV prevalence estimates from the Actuarial Society of South Africa Western Cape AIDS demographic model.^23^ We also calculated population disease rates relative to the year of test roll-out, overall and stratified by bacteriological confirmation. We estimated the population size for each year relative to test roll-out as follows, taking year 2 as an example: we calculated the proportion of all patients in each calendar year who were in year 2 of roll-out (Fig. 1) and multiplied this proportion by the estimated population for the corresponding calendar year. We then summed these estimates for all calendar years, which gave us the total estimated population for year 2 of roll-out.

Factors associated with empirical tuberculosis treatment, both overall and stratified by HIV status, were identified by multivariable logistic regression analysis. A priori risk factors included age, sex, HIV status, CD4+ cell count at the start of tuberculosis treatment, history of tuberculosis treatment, calendar year and year relative to test roll-out. Age, calendar year and year relative to test roll-out were included as either continuous or categorical variables based on the results of tests for departure from linearity. Because of the collinearity between our two-time variables, we used two separate multivariable logistic regression models – one included the calendar year and the other included the year relative to test roll-out. We accounted for clustering at the clinic level by calculating robust standard errors. In addition, a sensitivity analysis was performed using random effects models that adjusted for clustering at the clinic level. We tested for changes over time in the odds of empirical treatment in years 2, 3 and 4 of test roll-out using a model that included only those years. All analyses were performed using Stata/IC version 13.0 (StataCorp. LP, College Station, USA) and Excel 2013 (Microsoft Corporation, Redmond, USA). The study was approved by the Human Research Ethics Committee at the University of Cape Town and by the City of Cape Town Health Department.

## Results

In 2010, 21 255 patients with pulmonary tuberculosis aged 15 years or older were treated in Cape Town. The number declined annually to 19 174 in 2014, the year after the test roll-out was completed. Table 1 shows the patients’ demographic and clinical characteristics in each calendar year: their mean age was 35 years, 57% (57 664/102 007) were male and 48% (47 542/100 021) were HIV-positive. The HIV status was reported in 98% (100 021/102 007) of the patients. The only characteristic that changed substantially over time was the proportion of patients previously treated for tuberculosis, which was lower in later years. By March 2012, 62% (63/101) of tuberculosis clinics had access to the test. There was no difference in patients’ demographic or clinical characteristics by either year relative to test roll-out or calendar year. Details of the patients covered by rapid testing in each year and their characteristics are available from the corresponding author on request.

**Table 1:** Patients’ characteristics, impact of the rapid diagnostic test roll-out on tuberculosis diagnosis, Cape Town, South Africa, 2010–2014

Characteristic	Number of patients (%)^a^				
	Calendar year				
	2010	2011	2012	2013	2014
**All**	21 255	20 828	20 657	20 093	19 174
**Female**	9 602 (45)	9 214 (44)	8 883 (43)	8 621 (43)	8 023 (42)
**Age in years**					
15–24	3 991 (19)	3 728 (18)	3 654 (18)	3 487 (17)	3 287 (17)
25–34	7 099 (33)	6 756 (32)	6 574 (32)	6 472 (32)	6 046 (32)
35–44	5 271 (25)	5 461 (26)	5 425 (26)	5 315 (27)	4 991 (26)
45–54	3 202 (15)	3 171 (15)	3 236 (16)	3 053 (15)	3 130 (16)
55–64	1 222 (6)	1 235 (6)	1 286 (6)	1 249 (6)	1 233 (6)
≥65	470 (2)	477 (2)	482 (2)	517 (3)	487 (3)
**Previously treated for tuberculosis**	6 626 (31)	6 588 (32)	6 714 (33)	5 738 (29)	4 864 (25)
**HIV-positive^b^**	9 985 (48)	9 922 (49)	9 650 (48)	9 162 (46)	8 823 (47)
**CD4+ T-lymphocyte count per mm^3^, median (IQR)^c^**	167 (77–296)	181 (83–316)	185 (80–329)	179 (75–330)	173 (73–339)

HIV: human immunodeficiency virus; IQR: interquartile range; SD: standard deviation.

^a^ All values in the table represent absolute numbers and percentages unless otherwise stated.

^b^ The number of patients whose HIV status was unknown was 627 in 2010, 413 in 2011, 412 in 2012, 272 in 2013 and 262 in 2014.

^c^ The number of patients whose CD4+ T-lymphocyte count was unknown was 358 in 2010, 259 in 2011, 310 in 2012, 287 in 2013 and 584 in 2014.

Note: The roll-out of the automated Xpert® MTB/RIF test (Cepheid, Sunnyvale, United States of America) occurred between August 2011 and February 2013.

**Fig. 2:**
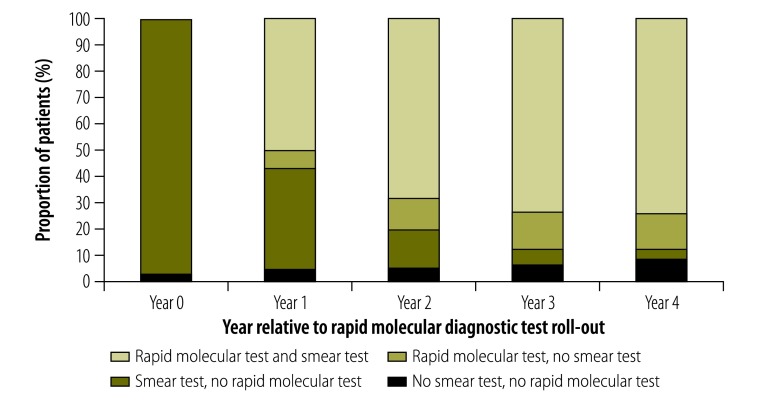
Diagnostic tests for pulmonary tuberculosis, by year relative to rapid diagnostic test roll-out, Cape Town, South Africa, 2010–2014

Notes: Patients who initiated tuberculosis treatment in the year before the Xpert® MTB/RIF test (Cepheid, Sunnyvale, United States of America) roll-out date for their clinic were assigned to year 0, those who started treatment in the first year after roll-out for their clinic were assigned to year 1 and so on up to year 4 (Fig. 1). The proportion of patients for whom only a sputum culture result was available was negligible.

The pattern of microbiological testing in patients with pulmonary tuberculosis changed during the test roll-out: use of this test increased to 88% (16 892/19 174) of patients in 2014, with 14 551 of the 16 892 (86%) testing positive. Correspondingly, utilization of sputum smear microscopy and sputum culture decreased. Fig. 2 shows that use of the test stabilized after the first 2 years of roll-out. Findings were similar among HIV-negative and HIV-positive patients (details available from the corresponding author on request).

The reasons for starting tuberculosis treatment changed over time: in 2010, the main reason was a positive sputum smear result in 67% (7100/10 643) of HIV-negative patients and in 41% (4082/9985) of HIV-positive patients; in 2014, the main reason was a positive Xpert® test result in 84% (8431/10 089) and 67% (5947/8823) of these patient groups, respectively (Table 2). Between 2010 and 2014, the proportion treated empirically decreased by 12 percentage points among HIV-negative patients and by 15 percentage points among HIV-positive patients. After excluding those for whom a positive sputum culture result became available after treatment initiation, the decrease in empirical treatment was 8 percentage points in both groups: among HIV-negative patients, the proportion decreased from 19% (2009/10 643) in 2010 to 11% (1115/10 089) in 2014; and, among HIV-positive patients, it decreased from 34% (3440/9985) to 26% (2293/8823). The proportion of patients with pulmonary tuberculosis diagnosed using the Xpert® test increased continuously during roll-out up to year 3 and stabilized thereafter (Table 3). The principal change underlying the decrease in empirical treatment during the study period was that fewer patients with a negative smear result were treated: among HIV-negative patients, 8% (800/10 643) were treated despite a negative smear result in 2010 compared with 1% (36/10 089) in 2014; among HIV-positive patients, the corresponding figures were 15% (1544/9985) and 1% (84/8823), respectively. In contrast, the proportion treated despite a negative Xpert® test result did not change substantially over time relative to test roll-out and the proportion treated because of abnormal chest X-ray findings alone decreased slightly among HIV-negative patients but did not change among HIV-positive patients, by both time definitions (details available from the corresponding author on request).

**Table 2:** Reason for tuberculosis treatment before, during and after the roll-out of a rapid diagnostic test for tuberculosis, Cape Town, South Africa, 2010–2014

Reason for starting tuberculosis treatment	Number of patients (%)				
	Calendar year				
	2010	2011	2012	2013	2014
**HIV-negative patients**					
Total^a^	10 643	10 493	10 595	10 659	10 089
Positive rapid test result	0	631 (6)	3 954 (37)	7 975 (75)	8 431 (84)
Positive sputum smear	7 100 (67)	6 623 (63)	4 040 (38)	1 012 (9)	452 (4)
Positive sputum culture	1 098 (10)	994 (9)	514 (5)	144 (1)	57 (1)
Empirical treatment^b^	2 445 (23)	2 245 (21)	2 087 (20)	1 528 (14)	1 149 (11)
**HIV-positive patients**					
Total^a^	9 985	9 922	9 650	9 162	8 823
Positive rapid test result	1 (0)	400 (4)	2 906 (30)	5 386 (59)	5 947 (67)
Positive sputum smear	4 082 (41)	3 693 (37)	2 290 (24)	658 (7)	248 (3)
Positive sputum culture	1 673 (17)	1 643 (17)	832 (9)	320 (3)	264 (3)
Empirical treatment^b^	4 229 (42)	4 186 (42)	3 622 (38)	2 798 (31)	2 364 (27)

HIV: human immunodeficiency virus.

^a^ The number of patients whose HIV status was unknown was 627 in 2010, 413 in 2011, 412 in 2012, 272 in 2013 and 262 in 2014.

^b^ Treatment was empirical when no test gave a positive result or no test was performed.

Note: The roll-out of the automated Xpert® MTB/RIF test (Cepheid, Sunnyvale, United States of America) occurred between August 2011 and February 2013.

**Table 3:** Reason for tuberculosis treatment, by year relative to the rapid diagnostic test roll-out, Cape Town, South Africa, 2010–2014

Reason for starting tuberculosis treatment	Number of patients (%)				
	Year relative to rapid test roll-out^a^				
	0	1	2	3	4
**HIV-negative patients**					
Total^b^	10 553	10 827	10 610	7 207	918
Positive rapid test result	0	6 045 (56)	8 141 (77)	5 976 (83)	766 (83)
Positive sputum smear	7 189 (68)	2 532 (23)	888 (8)	323 (4)	29 (3)
Positive sputum culture	1 002 (9)	372 (3)	124 (1)	37 (1)	8 (1)
Empirical treatment^c^	2 362 (22)	1 878 (17)	1 457 (14)	871 (12)	115 (13)
**HIV-positive patients**					
Total^b^	9 848	9 821	9 138	6 463	936
Positive rapid test result	0	4 177 (43)	5 494 (60)	4 297 (66)	630 (67)
Positive sputum smear	4 036 (41)	1 619 (16)	617 (7)	165 (3)	18 (2)
Positive sputum culture	1 636 (17)	726 (7)	310 (3)	165 (3)	22 (2)
Empirical treatment^c^	4 176 (42)	3 299 (34)	2 717 (30)	1 836 (28)	266 (28)

HIV: human immunodeficiency virus.

^a^ Patients who initiated tuberculosis treatment in the year before the Xpert® MTB/RIF test roll-out date for their clinic were assigned to year 0, those who started treatment in the first year after roll-out for their clinic were assigned to year 1 and so on up to year 4 (Fig. 1).

^b^ The number of patients whose HIV status was unknown was 627 in 2010, 413 in 2011, 412 in 2012, 272 in 2013 and 262 in 2014.

^c^ Treatment was empirical when no test gave a positive result or no test was performed.

Note: The roll-out of the automated Xpert® MTB/RIF test (Cepheid, Sunnyvale, United States of America) occurred between August 2011 and February 2013.

**Fig. 3:**
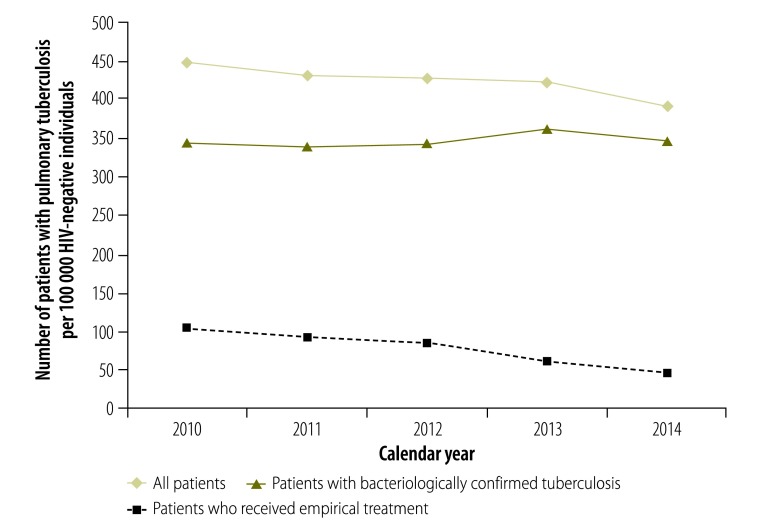
Tuberculosis notification rates in HIV-negative patients, by calendar year, Cape Town, South Africa, 2010–2014

**Fig. 4:**
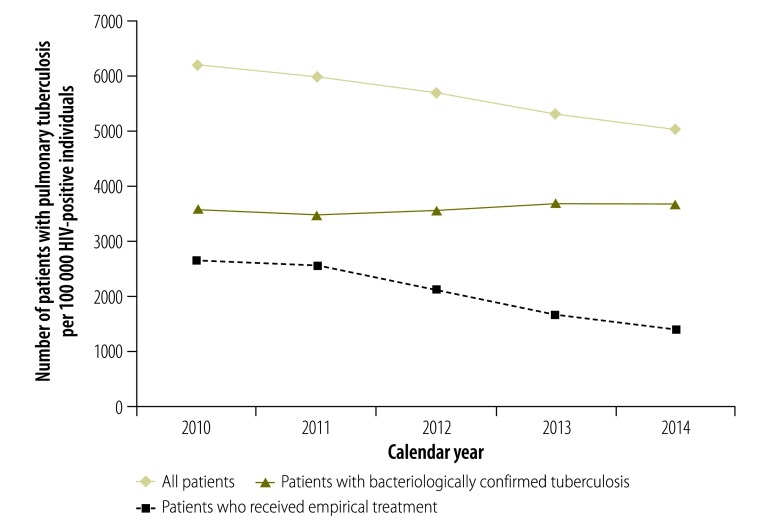
Tuberculosis notification rates in HIV-positive patients, by calendar year, Cape Town, South Africa, 2010–2014

**Fig. 5:**
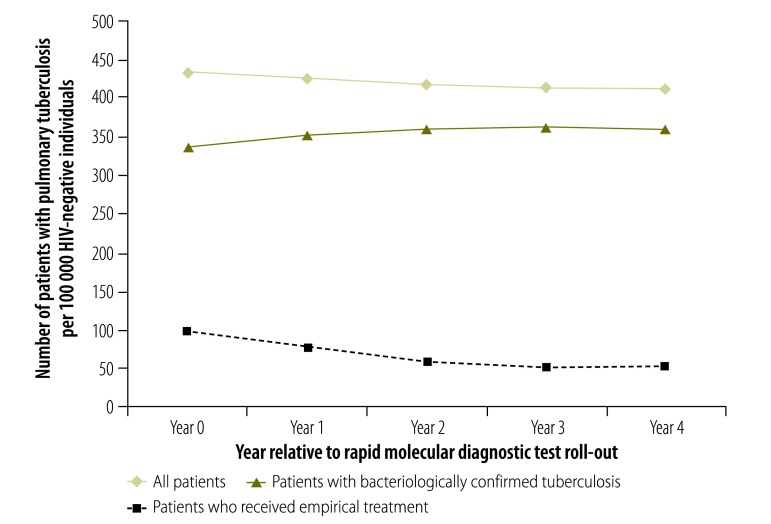
Tuberculosis notification rates in HIV-negative patients, by year relative to rapid diagnostic test roll-out, Cape Town, South Africa, 2010–2014

**Fig. 6:**
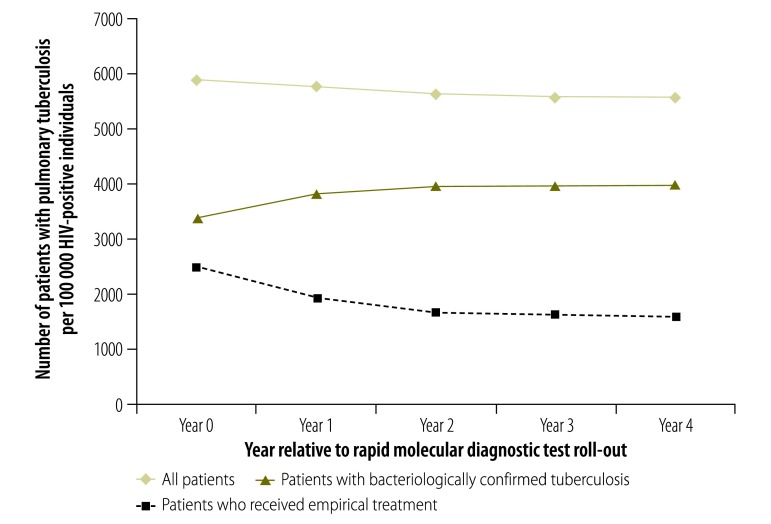
Tuberculosis notification rates in HIV-positive patients, by year relative to rapid diagnostic test roll-out, Cape Town, South Africa, 2010–2014

The tuberculosis notification rate in the adult population decreased over the 5-year study period: by 12% among HIV-negative individuals and by 19% among HIV-positive individuals (Fig. 3 and Fig. 4, respectively). The rate of bacteriologically confirmed tuberculosis increased by 1% and 3% in these two groups, respectively, and the rate of empirical treatment decreased by 56% and 49%, respectively. A slightly different pattern was seen when the data were analysed by year relative to test roll-out: the rate of bacteriologically confirmed tuberculosis increased between year 0 and year 4 by 7% in HIV-negative individuals (Fig. 5) and by 17% in HIV-positive individuals (Fig. 6), and the rate of empirical treatment decreased by 47% and 37%, respectively. These changes stabilized after year 3.

HIV: human immunodeficiency virus.

Note: Treatment was empirical when no test gave a positive result or no test was performed.

HIV: human immunodeficiency virus.

Note: Treatment was empirical when no test gave a positive result or no test was performed.

HIV: human immunodeficiency virus.

Notes: Patients who initiated tuberculosis treatment in the year before the Xpert® MTB/RIF test (Cepheid, Sunnyvale, United States of America) roll-out date for their clinic were assigned to year 0, those who started treatment in the first year after roll-out for their clinic were assigned to year 1 and so on up to year 4 (Fig. 1). Treatment was empirical when no test gave a positive result or no test was performed.

HIV: human immunodeficiency virus.

Notes: Patients who initiated tuberculosis treatment in the year before the Xpert® MTB/RIF test (Cepheid, Sunnyvale, United States of America) roll-out date for their clinic were assigned to year 0, those who started treatment in the first year after roll-out for their clinic were assigned to year 1 and so on up to year 4 (Fig. 1). Treatment was empirical when no test gave a positive result or no test was performed.

Multivariable logistic regression analysis showed that the odds of empirical tuberculosis treatment were 2.75-fold higher in HIV-positive than HIV-negative patients (adjusted odds ratio: 2.75; 95% confidence interval: 2.55–2.98). Other factors associated with empirical treatment were all patients older than 45 years and female sex in HIV-infected patients. After adjusting for these factors, the odds of empirical treatment decreased with time relative to test roll-out (Table 4). There was no evidence to support a further reduction in odds between years 2, 3 and 4 (*P* = 0.22). When the analysis was performed separately in HIV-positive and HIV-negative patients, the same risk factors were identified (Table 4). Among HIV-positive patients, every 50-cells/mm^3^ increase in CD4+ cell count at tuberculosis diagnosis was associated with 4% lower odds of empirical treatment. These results were found to be robust in the sensitivity analysis performed using a random effects model (details available from the corresponding author on request).

**Table 4:** Risk factors associated with empirical tuberculosis treatment, Cape Town, South Africa, 2010–2014

Risk factor	Risk of empirical treatment,^a^ by multivariable logistic regression analysis					
	HIV-negative patients		HIV-positive patients		All patients	
	aOR (95% CI)	*P*-value	aOR (95% CI)	*P*-value	aOR (95% CI)	*P*-value
**Sex**		0.92		< 0.001		0.15
Female	Reference		Reference		Reference	
Male	1.00 (0.92–1.08)		0.92 (0.88–0.97)		0.97 (0.93–1.01)	
**Age in years**		< 0.001		< 0.001		< 0.001
15–24	Reference		Reference		Reference	
25–34	0.93 (0.83–1.04)		1.13 (1.04–1.22)		1.04 (0.96–1.12)	
35–44	1.02 (0.92–1.14)		1.22 (1.12–1.34)		1.12 (1.03–1.22)	
45–54	1.32 (1.18–1.47)		1.37 (1.22–1.54)		1.31 (1.19–1.44)	
55–64	1.77 (1.51–2.07)		1.44 (1.23–1.68)		1.64 (1.45–1.87)	
≥ 65	2.91 (2.56–3.31)		2.33 (1.65–3.29)		2.89 (2.55–3.27)	
**CD4+ T-lymphocyte count, per 50-cells/mm^3^ increase**	N/A	N/A	0.96 (0.96–0.97)	< 0.001	N/A	NA
**Prior tuberculosis treatment**		0.54		< 0.001		0.02
No	Reference		Reference		Reference	
Yes	0.97 (0.87–1.07)		1.20 (1.11–1.30)		1.09 (1.02–1.18)	
**Year relative to rapid test roll-out^b^**		< 0.001^c^		< 0.001^c^		< 0.001^c^
0	Reference		Reference		Reference	
1	0.73 (0.64–0.82)		0.69 (0.63–0.77)		0.70 (0.64–0.77)	
2	0.55 (0.48–0.61)		0.58 (0.51–0.65)		0.56 (0.51–0.62)	
3	0.47 (0.41–0.54)		0.55 (0.47–0.64)		0.51 (0.45–0.58)	
4	0.48 (0.32–0.71)		0.54 (0.41–0.72)		0.52 (0.39–0.68)	

aOR: adjusted odds ratio; CI: confidence interval; HIV: human immunodeficiency virus; N/A: not applicable.

^a^ Treatment was empirical when no test gave a positive result or no test was performed.

^b^ Patients who initiated tuberculosis treatment in the year before the Xpert® MTB/RIF test (Cepheid, Sunnyvale, United States of America) roll-out date for their clinic were assigned to year 0, those who started treatment in the first year after roll-out for their clinic were assigned to year 1 and so on up to year 4 (Fig. 1).

^c^ *P*-values calculated using the Wald test were also < 0.001 for each roll-out year relative to year 0.

## Discussion

We found that the introduction of the Xpert® test as the first-line diagnostic test for tuberculosis in a large population cohort led to this test becoming the primary method of diagnosis in three quarters of adults treated for drug-sensitive pulmonary tuberculosis. In addition, the rate of bacteriologically confirmed disease increased following the introduction of the test and the rate of empirical treatment decreased, resulting in a net decline in the total notification rate for pulmonary tuberculosis. These changes occurred cumulatively with test roll-out and stabilized after 3 years.

Few evaluations of the impact of routine Xpert® testing in programmatic settings have been published and most documented difficulties with roll-out and implementation.^24^^–^^29^ Increased proportions of bacteriological confirmation and less empirical treatment were reported in Nepal and India but an increase in the case notification rate was reported only in India.^30^^,^^31^ Moreover, the four large randomized clinical trials performed to date all reported increased proportions of bacteriological confirmation but only one, performed in Cape Town,^11^ found that the number of patients diagnosed with tuberculosis increased.^10^^–^^13^

Our data from a programmatic setting in Cape Town are consistent with previous findings: routine use of the rapid test did not lead to an increase in the tuberculosis notification rate but was temporally associated with an increased bacteriological confirmation rate and a decrease in empirical treatment. The net effect was an apparent decline in the total tuberculosis notification rate. We previously reported that notification rates have decreased since 2010 in both HIV-negative and HIV-positive individuals.^32^ There are two potential, complementary explanations: (i) the incidence of tuberculosis decreased (assuming access to diagnosis did not change); and (ii) empirical treatment decreased. It was not possible to separate the contributions of these factors using our data. However, the observation that the rate of bacteriologically confirmed tuberculosis remained stable with the increasing use of a more sensitive test suggests that the incidence of tuberculosis may have decreased. A possible underlying mechanism could be greater use of antiretroviral therapy in the HIV-infected population – coverage increased from 0% in 2004 to 63% in 2013 in Cape Town.^32^ However, the decline in tuberculosis notification rates we observed was not affected by HIV status, which does not support this explanation. In contrast, the possibility that the empirical treatment rate decreased with the test roll-out is supported by our observation that the decline in this rate slowed gradually during roll-out and then stabilized. This may reflect the time needed to fully implement the new test and, possibly, to apply the new diagnostic algorithm. If the decrease in the tuberculosis notification rate we observed were mainly attributable to a reduction in empirical treatment, we would expect the decline to stabilize within 3 to 4 years of the test being introduced at the last clinic (i.e. by the end of 2017). If the decline continues thereafter, it is probably attributable to another factor, such as declining incidence.^12^

The main limitation of our study was its inability to determine whether the decline in empirical treatment represented a decline in false-positive or true-positive diagnoses. The latter could have occurred because clinicians overestimated the negative predictive value of the rapid test. Interestingly, the proportional decline in empirical treatment was smaller among HIV-infected patients, in whom the test is less sensitive.^3^ Clinicians may have been reluctant to miss active tuberculosis disease in this vulnerable population, thereby increasing the number of false-positive diagnoses. Our lack of data on presumptive tuberculosis patients precluded an evaluation of whether roll-out of the test led to the identification and treatment of patients who would otherwise not have been treated. Moreover, we were not able to investigate the impact of symptomatology on the likelihood of empirical treatment (no data) or of the time to treatment initiation (incomplete recording of sputum collection dates). The apparent decrease in previously treated patients was probably due to misclassification following abolition of the distinct retreatment regimen.

In conclusion, routine use of the Xpert® test in a setting with a high prevalence of tuberculosis and HIV infection was associated with a halving of the empirical treatment rate. This reduction occurred gradually following the introduction of the test, probably due to the time needed for full implementation of a new diagnostic algorithm. More than a quarter of HIV-infected patients were still treated empirically, which highlights the difficulty of diagnosing tuberculosis in this group.

## References

[1] Boehme CC, Nicol MP, Nabeta P, Michael JS, Gotuzzo E, Tahirli R, et al. Feasibility, diagnostic accuracy, and effectiveness of decentralised use of the Xpert MTB/RIF test for diagnosis of tuberculosis and multidrug resistance: a multicentre implementation study. Lancet. 2011 4 30;377(9776):1495–505. 10.1016/S0140-6736(11)60438-8 21507477 PMC3085933

[2] Boehme CC, Nabeta P, Hillemann D, Nicol MP, Shenai S, Krapp F, et al. Rapid molecular detection of tuberculosis and rifampin resistance. N Engl J Med. 2010 9 9;363(11):1005–15. 10.1056/NEJMoa0907847 20825313 PMC2947799

[3] Steingart KR, Schiller I, Horne DJ, Pai M, Boehme CC, Dendukuri N. Xpert® MTB/RIF assay for pulmonary tuberculosis and rifampicin resistance in adults. Cochrane Database Syst Rev. 2014 1 21;1(1):CD009593. 24448973 10.1002/14651858.CD009593.pub3 PMC4470349

[4] WHO endorses new rapid tuberculosis test: a major milestone for global TB diagnosis and care. News release. Geneva: World Health Organization; 2010. Available from: http://www.who.int/mediacentre/news/releases/2010/tb_test_20101208/en/ [cited 2014 Dec 15].

[5] Albert H, Nathavitharana RR, Isaacs C, Pai M, Denkinger CM, Boehme CC. Development, roll-out and impact of Xpert MTB/RIF for tuberculosis: what lessons have we learnt and how can we do better? Eur Respir J. 2016 8;48(2):516–25. 10.1183/13993003.00543-2016 27418550 PMC4967565

[6] Priorities for tuberculosis research: a report of the disease reference group report on TB, leprosy and Buruli ulcer. Geneva: World Health Organization; 2013. Available from: http://apps.who.int/iris/bitstream/10665/85888/1/9789241505970_eng.pdf. [cited 2017 Apr 18].

[7] Menzies NA, Cohen T, Lin HH, Murray M, Salomon JA. Population health impact and cost-effectiveness of tuberculosis diagnosis with Xpert MTB/RIF: a dynamic simulation and economic evaluation. PLoS Med. 2012;9(11):e1001347. 10.1371/journal.pmed.1001347 23185139 PMC3502465

[8] Meyer-Rath G, Schnippel K, Long L, MacLeod W, Sanne I, Stevens W, et al. The impact and cost of scaling up GeneXpert MTB/RIF in South Africa. PLoS One. 2012;7(5):e36966. 10.1371/journal.pone.0036966 22693561 PMC3365041

[9] Dowdy DW, Davis JL, den Boon S, Walter ND, Katamba A, Cattamanchi A. Population-level impact of same-day microscopy and Xpert MTB/RIF for tuberculosis diagnosis in Africa. PLoS One. 2013 8 12;8(8):e70485. 10.1371/journal.pone.0070485 23950942 PMC3741313

[10] Theron G, Zijenah L, Chanda D, Clowes P, Rachow A, Lesosky M, et al.; TB-NEAT team. Feasibility, accuracy, and clinical effect of point-of-care Xpert MTB/RIF testing for tuberculosis in primary-care settings in Africa: a multicentre, randomised, controlled trial. Lancet. 2014 2 1;383(9915):424–35. 10.1016/S0140-6736(13)62073-5 24176144

[11] Cox HS, Mbhele S, Mohess N, Whitelaw A, Muller O, Zemanay W, et al. Impact of Xpert MTB/RIF for TB diagnosis in a primary care clinic with high TB and HIV prevalence in South Africa: a pragmatic randomised trial. PLoS Med. 2014 11 25;11(11):e1001760. 10.1371/journal.pmed.1001760 25423041 PMC4244039

[12] Durovni B, Saraceni V, van den Hof S, Trajman A, Cordeiro-Santos M, Cavalcante S, et al. Impact of replacing smear microscopy with Xpert MTB/RIF for diagnosing tuberculosis in Brazil: a stepped-wedge cluster-randomized trial. PLoS Med. 2014 12 9;11(12):e1001766. 10.1371/journal.pmed.1001766 25490549 PMC4260794

[13] Churchyard GJ, Stevens WS, Mametja LD, McCarthy KM, Chihota V, Nicol MP, et al. Xpert MTB/RIF versus sputum microscopy as the initial diagnostic test for tuberculosis: a cluster-randomised trial embedded in South African roll-out of Xpert MTB/RIF. Lancet Glob Health. 2015 8;3(8):e450–7. 10.1016/S2214-109X(15)00100-X 26187490

[14] Theron G, Peter J, Dowdy D, Langley I, Squire SB, Dheda K. Do high rates of empirical treatment undermine the potential effect of new diagnostic tests for tuberculosis in high-burden settings? Lancet Infect Dis. 2014 6;14(6):527–32. 10.1016/S1473-3099(13)70360-8 24438820

[15] Menzies NA, Cohen T, Murray M, Salomon JA. Effect of empirical treatment on outcomes of clinical trials of diagnostic assays for tuberculosis. Lancet Infect Dis. 2015 1;15(1):16–7. 10.1016/S1473-3099(14)71026-6 25541164

[16] Sun AY, Denkinger CM, Dowdy DW. The impact of novel tests for tuberculosis depends on the diagnostic cascade. Eur Respir J. 2014 11;44(5):1366–9. 10.1183/09031936.00111014 25186263 PMC4254765

[17] Management and treatment of TB in adults and children older than 8 years. Circular No: H22/2013. Cape Town: Western Cape Department of Health; 2013.

[18] City of Cape Town – 2011 Census – Cape Town. Cape Town: Strategic Development Information and GIS Department, City of Cape Town; 2011. Available from: http://resource.capetown.gov.za/documentcentre/Documents/Maps%20and%20statistics/2011_Census_Cape_Town_Profile.pdf [cited 2017 Apr 18].

[19] Stevens M, Sinanovic E, Regensberg L, Hislop M. HIV and AIDS, STI and TB in the private sector. Chapter 14. In: South African Health Review 2007. Durban: Health Systems Trust; 2007. Available from: http://www.hst.org.za/uploads/files/chap14_07.pdf [cited 2017 Jan 30].

[20] Whitelaw A, Sturm W. Microbiological testing for *Mycobacterium tuberculosis*. In: Schaaf H, Zumla A, editors. Tuberculosis: a comprehensive reference. London: Saunders; 2009.

[21] National tuberculosis management guidelines 2009. Pretoria: National Department of Health, Republic of South Africa; 2009. Available from: http://familymedicine.ukzn.ac.za/Libraries/Guidelines_Protocols/TB_Guidelines_2009.sflb.ashx [cited 2013 Nov 04].

[22] District council projections 2002–2016 [spreadsheet]. Pretoria: Statistics South Africa, National Department of Health, Health Information Systems Programme; 2010. Available from: http://www.statssa.gov.za/publications/P0302/District_Council_projection_by_sex_and_age_(2002-2016).xlsx [cited 2017 Apr 18].

[23] AIDS models [Internet]. Cape Town: Actuarial Society of South Africa; 2008. Available from: http://www.actuarialsociety.org.za/Societyactivities/CommitteeActivities/DemographyEpidemiologyCommittee/Models.aspx [cited 2012 Jul 17].

[24] Creswell J, Codlin AJ, Andre E, Micek MA, Bedru A, Carter EJ, et al. Results from early programmatic implementation of Xpert MTB/RIF testing in nine countries. BMC Infect Dis. 2014 1 2;14(1):2. 10.1186/1471-2334-14-2 24383553 PMC3898850

[25] Durovni B, Saraceni V, Cordeiro-Santos M, Cavalcante S, Soares E, Lourenço C, et al. Operational lessons drawn from pilot implementation of Xpert MTB/Rif in Brazil. Bull World Health Organ. 2014 8 1;92(8):613–7. 10.2471/BLT.13.131409 25177076 PMC4147406

[26] Cowan J, Michel C, Manhiça I, Monivo C, Saize D, Creswell J, et al. Implementing rapid testing for tuberculosis in Mozambique. Bull World Health Organ. 2015 2 1;93(2):125–30. 10.2471/BLT.14.138560 25883406 PMC4339961

[27] Sikhondze W, Dlamini T, Khumalo D, Maphalala G, Dlamini S, Zikalala T, et al. Countrywide roll-out of Xpert(®) MTB/RIF in Swaziland: the first three years of implementation. Public Health Action. 2015 6 21;5(2):140–6. 10.5588/pha.15.0001 26400386 PMC4487476

[28] Molapo S, Berrie L, Marokane P, Magida V, Scott L, Stevens W. GeneXpert module failures: South Africa’s Xpert MTB/RIF national programme experience and impact on costs. Int J Tuberc Lung Dis. 2014 11;18(11) suppl 1:s395 Available from: http://barcelona.worldlunghealth.org/programme/body/Abstract_Book_2014-Web.pdf [cited 2017 Apr 18].

[29] Hanrahan CF, Haguma P, Ochom E, Kinera I, Cobelens F, Cattamanchi A, et al. Implementation of Xpert MTB/RIF in Uganda: missed opportunities to improve diagnosis of tuberculosis. Open Forum Infect Dis. 2016 5 12;3(2):ofw068. 10.1093/ofid/ofw068 27186589 PMC4866550

[30] Creswell J, Rai B, Wali R, Sudrungrot S, Adhikari LM, Pant R, et al. Introducing new tuberculosis diagnostics: the impact of Xpert(®) MTB/RIF testing on case notifications in Nepal. Int J Tuberc Lung Dis. 2015 5;19(5):545–51. 10.5588/ijtld.14.0775 25868022

[31] Sachdeva KS, Raizada N, Sreenivas A, Van’t Hoog AH, van den Hof S, Dewan PK, et al. Use of Xpert MTB/RIF in decentralized public health settings and its effect on pulmonary TB and DR-TB case finding in India. PLoS One. 2015 5 21;10(5):e0126065. 10.1371/journal.pone.0126065 25996389 PMC4440647

[32] Hermans S, Boulle A, Caldwell J, Pienaar D, Wood R. Temporal trends in TB notification rates during ART scale-up in Cape Town: an ecological analysis. J Int AIDS Soc. 2015 9 25;18(1):20240. 10.7448/IAS.18.1.20240 26411694 PMC4584214

